# Efficacy of CAR-T immunotherapy in MET overexpressing tumors not eligible for anti-MET targeted therapy

**DOI:** 10.1186/s13046-022-02479-y

**Published:** 2022-10-21

**Authors:** Cristina Chiriaco, Chiara Donini, Marco Cortese, Stefano Ughetto, Chiara Modica, Ilaria Martinelli, Alessia Proment, Letizia Vitali, Lara Fontani, Monica Casucci, Paolo Maria Comoglio, Silvia Giordano, Dario Sangiolo, Valeria Leuci, Elisa Vigna

**Affiliations:** 1 Candiolo Cancer Institute, FPO-IRCCS, Strada Provinciale 142, 10060 Candiolo, TO Italy; 2 Present address: Anemocyte S.r.l., 21040 Gerenzano, VA Italy; 3 Department of Oncology, University of Turin, Turin, Italy; 4 Present address: Bios-Therapy, Physiological System for Health S.p.A, 52037 Sansepolcro, AR Italy; 5 Present address: Department of Surgical, Oncological and Stomatological Sciences (DICHIRONS), University of Palermo, 90127 Palermo, Italy; 6 Innovative Immunotherapies Unit, IRCCS San Raffaele Scientific Institute, Milan, Italy; 7 IFOM-FIRC Institute of Molecular Oncology, Milan, Italy

**Keywords:** MET oncogene, Immunotherapy, CAR, Targeted therapy, Gastric cancer

## Abstract

**Background:**

Aberrant activation of the MET receptor in cancer is sustained by genetic alterations or, more frequently, by transcriptional upregulations. A fraction of MET-amplified or mutated tumors are sensible to MET targeting agents, but their responsiveness is typically short-lasting, as secondary resistance eventually occurs. Since in the absence of genetic alterations MET is usually not a tumor driver, MET overexpressing tumors are not/poorly responsive to MET targeted therapies. Consequently, the vast majority of tumors exhibiting MET activation still represent an unmet medical need.

**Methods:**

Here we propose an immunotherapy strategy based on T lymphocytes expressing a Chimeric Antigen Receptor (CAR) targeting MET overexpressing tumors of different histotypes. We engineered two different MET-CAR constructs and tested MET-CAR-T cell cytotoxic activity against different MET overexpressing models, including tumor cell lines, primary cancer cells, organoids, and xenografts in immune-deficient mice.

**Results:**

We proved that MET-CAR-T exerted a specific cytotoxic activity against MET expressing cells. Cell killing was proportional to the level of MET expressed on the cell surface. While CAR-T cytotoxicity was minimal versus cells carrying MET at physiological levels, essentially sparing normal cells, the activity versus MET overexpressing tumors was robust, significantly controlling tumor cell growth in vitro and in vivo. Notably, MET-CAR-T cells were also able to brake acquired resistance to MET targeting agents in MET amplified cancer cells carrying secondary mutations in downstream signal transducers.

**Conclusions:**

We set and validated at the pre-clinical level a MET-CAR immunotherapy strategy potentially beneficial for cancers not eligible for MET targeted therapy with inhibitory molecules, including those exhibiting primary or secondary resistance.

**Supplementary Information:**

The online version contains supplementary material available at 10.1186/s13046-022-02479-y.

## Background

The MET gene product, a receptor tyrosine kinase, stands out as one of the most important oncogenes activated in cancer. Upon paracrine stimulation by its specific ligand – the Hepatocyte Growth Factor – MET controls a network of intracellular signals including pro-mitogenic, pro-invasive, and anti-apoptotic cues, essential during embryogenesis and tissue regeneration [1, 2]. The same signals, when aberrantly activated, are crucial during cell transformation. Dysregulation of MET activation results from receptor gene alterations (amplification, point mutations, or translocations) or transcriptional upregulation, involving either the receptor – sustaining MET overexpression - or the ligand that, in case of ectopic expression, triggers autocrine loops [3, 4]. In well-defined genetic conditions – namely MET gene amplification and/or exon 14 skipping - MET acts as a ‘driver’ to which tumor cells are ‘addicted’ (*i.e.* MET is the oncogene that solely sustains transformation) [5–7]. In these cases, MET targeting molecules effectively counteract tumor growth; accordingly, data obtained from case reports and clinical trials demonstrate that a significant number of patients benefitted from anti-MET treatments [8–12]. MET gene amplification is also a molecular mechanism sustaining resistance to EGFR, HER-2 and BRAF targeted therapies in several cancer types. Also in these cases, MET targeted therapy improves the therapeutic outcome, bypassing the resistance [13–17]. Nevertheless, within the fraction of tumors responsive to anti-MET treatment, after the first period of effective response targeting molecules invariably favor the emergence of resistant clones eventually responsible for disease progression, substantially limiting the success of the therapy. This is a well-known condition due to tumor heterogeneity [18].

While MET gene amplification and exon 14 skipping are quite rare - less than 4% of total cancers - MET activation due to transcriptional overexpression is rather common, especially among carcinomas [19, 20]. Indeed, the complex intracellular response elicited by MET gives a better fitness to the tumor, helping to overcome selective barriers during cancer onset and progression [21, 22]. In line with this, MET gene overexpression is strongly associated with poor prognosis [23]. Considering solid tumors, MET overexpression can be considered a marker of transformation but, unfortunately, this feature does not help therapy, as *‘per se’* it does not confer MET addiction, resulting in a lack of effectiveness of classical targeting agents. Thus, the vast majority of tumors exhibiting MET activation cannot be included in protocols of MET-targeted therapy.

The transfer of ex vivo expanded T lymphocytes characterized by a redirected tumor reactivity by the expression of Chimeric Antigen Receptors (CARs) represents a highly promising approach in the field of cancer immunotherapy [24]. CARs are transmembrane synthetic molecules in which the extracellular portion, deputed to antigen recognition, selects for a cancer target, and the intracellular portion, deputed to the activation of specific intracellular signals, is responsible for the cytotoxic response. The extracellular portion includes commonly variable regions of an antibody formatted as a single chain, and the intracellular region comprises domains derived from the T cell receptor complex, *i.e.* the CD3ζ, that, upon activation by phosphorylation, elicits the cytotoxic response [25]. To further sustain the response, CD3ζ can be coupled to functional domains of a costimulatory molecule. The number of costimulatory domains defines the CAR type: the first generation, representing the ancestor CAR design, has CD3ζ only, the second generation includes one costimulatory domain, and the third generation has more than one domain derived from different costimulatory molecules. When expressed by T cells, CARs elicit a robust and sustained cytotoxic response against cells expressing the target molecule engaged by the CAR on their surface, independently from HLA/MHC presentation. Nowadays, CAR adoptive immunotherapy got impressive success in the eradication of hematological tumors [26], thus representing the elective choice for the clinical treatment of CD19 expressing B cell malignancies. In addition to this consolidated application, several studies are ongoing to potentiate the therapeutic outcome of CAR-T in more challenging cancer settings.

In this paper, we describe the design and validation at the pre-clinical level of a CAR-based immunotherapy strategy to hit MET overexpressing cancers, not eligible for MET targeting agents.

## Methods

### Cells

A549, NCI-H1993, NCI-H226, NCI-H441 human lung adenocarcinoma cells, Hs746T human gastric carcinoma cells, Caki-I kidney carcinoma cells, and 293T embryonic kidney cells were from the American Type Culture Collection (ATCC/LGC Standards Srl). EBC-1 human lung carcinoma cells were from the Japanese Collection of Research Bioresources. GTL-16 is a clone derived from MKN-45 human gastric carcinoma cells [27]. GTL-16_Res were obtained by prolonged treatment of GTL-16 cells with the anti-MET Tyrosine Kinase Inhibitor (TKI) PHA-665752 [28]. Cell lines were cultured according to the manufacturer’s instructions. GTR-164, GTR-498, and GTR-210 primary gastric carcinoma cells were derived from the respective Patient Derived Xenografts and maintained as described in [29]. L1.13 Cancer of Unknown Primary origin (CUP) primary cell line was derived and maintained as described in [15]. Huvec cells were obtained from dr. Gabriella Doronzo (University of Turin) and maintained as described in [30]. Primary human skin fibroblasts were obtained from Prof. Antonia Follenzi (University of Piemonte Orientale) and maintained as described in [31]. Primary human epidermal keratinocytes were from Lonza and maintained as suggested by the supplier.

### Generation of genetically modified A549 cells

A549_MET^+^: A549 cells were plated 50.000 cells/well in 6 well plates in complete medium. After 24 hrs, the medium was replaced and the cells were transduced with 400 ng/mL p24 of LV-TETMET, a lentiviral vector expressing MET wild-type under the control of tetracycline (TET)-inducible promoter (TET-off system) [32] with polybrene (8 μg/mL, Sigma-Aldrich) and doxycycline (50 ng/mL, Sigma-Aldrich). After transduction, cells were maintained in culture without doxycycline to induce MET expression.

A549_koMET: Single guides RNA (sgRNA) were designed on the second exon of the MET gene. Sequences were: i) forward CACCG GGTGTTTCCGCGGTGAAGTT; ii) reverse AAAC AACTTCACCGCGGAAACACC C. sgRNA were cloned in the Px456 plasmid (Addgene), containing Puromycin resistance and the Cas9 sequence. This plasmid was used to transfect A549 cells with the TransIT-X2 Dynamic Delivery System (Mirus) in accordance with the manufacturer’s protocol. Cells were cultured for 7 days with Puromycin (1 μg/mL, Sigma-Aldrich) and then were seeded in 96 well plates to obtain single clones limiting dilution. Knock-out of the MET gene was checked by gDNA sequencing.

### Generation of DO24 single-chain antibody formats

From the sequence of the DO24 monoclonal antibody [33] we designed: 1) a single-chain variable fragment (scFv) DO24 by inserting a linker of 25 aa between the variable light and variable heavy regions; 2) a single-chain FAb (scFAb) by inserting a linker of 60 aa between the light and the truncated heavy (Variable Heavy-Constant Heavy-1) chains. Constant regions were from human Ig*k* and IgG1 sequences, respectively. cDNA synthesis, protein expression in mammalian HEK293E cells, and purification of the single-chain antibody fragments were performed by U-Protein Express BV.

### Analysis of DO24 binding to MET

DO24 single-chain binding affinities were determined by ELISA assay; the MET extracellular domain fused in frame with a human Fc domain (100 ng/well, R&D System) was in the solid phase and increasing concentrations of the purified DO24 antibody formats (range 0–33 nM) were in the liquid phase. Binding was revealed using horseradish peroxidase (HRP)-conjugated anti-human k light chain antibodies (Sigma-Aldrich). The colorimetric signal was quantified by the multi-label reader VICTOR X4 (Perkin Elmer Instrument INC.). To calculate Kd and Bmax, data were analyzed and fitted according to nonlinear regression, one-site binding hyperbola curve, using GraphPad Prism software (GraphPad Software).

### MET-CAR lentiviral vector construction

DO24 scFv and scFAb were cloned as XmaI-SalI fragments in sense into a lentiviral vector (LV) with a bidirectional expression cassette carrying a cDNA encoding the eGFP (enhanced Green Fluorescent Protein) in the antisense position with respect to the internal promoter hPGK [34]. Then, a sequence (AgeI-MluI) obtained by PCR-amplification from a plasmid encoding for a CD44v6 CAR [35] was cloned in frame at the 3′ of the DO24-derived sequences. The amplified region corresponded to: 1) Hinge + Constant Heavy 2 and 3 domains, derived from human IgG1, transmembrane + intracellular domains derived from human CD28, and CD3ζ from human T Cell Receptor, in the case of DO24 scFv; 2) hinge domain derived from human IgG1, transmembrane + intracellular domains derived from human CD28, and CD3ζ from human T Cell Receptor, in the case of DO24 scFAb.

### Lentiviral vector particle preparation

LV stocks were obtained by transient transfection of 293T cells with 37.5 μg of transfer vector, 16.5 μg of the packaging plasmids pMDLg/pRRE, 6.25 μg of the plasmid pRSV.REV, and 9 μg of the vesicular stomatitis virus (VSV) envelope plasmid pMD2.VSV-G as described in [36]. Determination of the viral p24 antigen concentration and MET-CAR LVs’ titer were determined as described in [36].

### Isolation, activation, transduction, and expansion of MET-CAR-T

T cells were purified from peripheral blood mononuclear cells (PBMC) of healthy donors or of patients with gastric carcinoma. PBMC were isolated by density gradient centrifugation (Lymphosep, Aurogene) and then were activated by anti-Biotin MACSi Bead Particles loaded with anti-CD2, anti-CD3, and anti-CD28 antibodies (Miltenyi Biotec) for 24 hrs. PBMC were maintained in culture medium + human recombinant IL-2 (100 U/mL, Miltenyi Biotech) for a further 24 hrs and then transduced with MET-CAR-LVs (MOI 10) for 8 hrs in the presence of polybrene (8 μg/mL). Not-transduced T cells (NTD) were used as a paired control. CAR-T or NTD-T cells were expanded for a maximum of 3 weeks in the presence of IL-2 (100 U/mL). Transduction efficiency (evaluated by eGFP expression) ranged from 30 to 70% of the total population.

### Flow cytometry analysis

Primary antibodies: anti-MET (Human HGFR/c-MET APC-conjugated Antibody, clone 95,106, R&D System); anti-IgG1/CH2CH3 regions (Alexa Fluor® 647 AffiniPure F (ab’)2 Fragment Goat Anti-Human IgG (H + L) antibody, Jackson Immuno Research); anti-CD4 (APC Mouse Anti-Human CD4 clone M-T466, Miltenyi); anti-CD3 (PE Mouse Anti-Human CD3, Clone HIT3a); anti-CD8 (APC Mouse Anti-Human CD8, Clone RPA-T8); anti-CD56 (PE Mouse Anti-Human CD56, Clone MY31) all from BD Biosciences. Isotype control antibodies: APC, FITC, or PE mouse IgG1 κ Isotype Control, Clone MOPC-21 (BD Biosciences). Cells were counterstained with DAPI and analyzed by Cyan ADP flow cytometer (Beckman Coulter S.r.l.). Data were elaborated using Summit 4.3 software (Dako). For plots in which the isotype control is not shown, the Mean Fluorescence Intensity (MFI) derived from the Isotype control was set within the first logarithm (0 < MFI < 10). Cells were considered positive for the analyzed marker if the signal was higher than 10 (MFI > 10).

### Quantitative flow cytometry analysis of MET expression

For determination of MET expression, Quantum™ Simply Cellular® microbeads (Bangs Laboratories) were used to build a calibration curve to which cell samples have been compared. Cells of interest and calibration microbeads characterized by an increasing antibody binding capacity (ABC) were labeled simultaneously to saturation with the anti-MET antibody (Human HGFR/c-MET, clone 95,106, PE-conjugated Antibody, R&D Systems). A non-binding microbead population represents the negative control. Beads and labeled cells were acquired on Cyan ADP flow cytometer and analyzed using Summit 4.3 software. The obtained median values, subtracted of the median value of each relative isotype control, were used to calculate the ABC of each cell population, using the lot-specific QuickCal® analysis template provided by the supplier.

### In vitro cytotoxicity assays

Bioluminescent cell viability essay: Target cells (5000/well in a 96 well plate) were co-cultured with effector cells at various effectors/target (E:T) ratios (5:1, 2.5:1, 1:1, 1:2 and 1:4) for 24 hours (Hs746T, EBC-1, GTL-16, Caki-1, GTR-164, GTR-498, GTR-210, and GTL-16_Res cells) or 48 hours (A549, 293 T, Huvec, primary keratinocytes, primary fibroblasts, NCI-H1993, NCI-H226, NCI-H-441, and L1.L13 cells) in culture medium with IL-2 (100 U/mL). Cell viability was determined by Cell Titer-Glo Luminescent Cell Viability Assay (Promega). The chemiluminescent signal was detected with VICTOR X4. To determine MET-CAR-T killing specificity, target cells were pre-incubated and co-cultured with 1 μM of decoyMET [37] produced and purified by U-Protein Express BV. To confirm the results, in selected experiments, cytotoxicity was also evaluated by a flow-cytometry-based essay labelling the cells with the vital dye PKH26 (Sigma-Aldrich) as described in [38]. The percentage of tumor-specific lysis for each E:T ratio was calculated using the following formula: [(experimental − spontaneous mortality/100 − spontaneous mortality) × 100].

### Immunofluorescence analysis of MET-CAR-T recruitment and infiltration in 3D organoids

GTR-498-derived organoids were plated in Matrigel (BD Pharmingen) domes in 8-well glass-bottom chamber slides (Falcon). After 48–72 hours organoids were overnight labeled with NucBlue (NucBlue™ Live Ready Probes™ Reagent) directly in the culture chamber slide wells. Then GTR-498-derived organoids were co-cultured with MET-CAR T or paired NTD cells stained with PKH26 dye at an E:T ratio of 2:1 in culture medium in the presence of IL-2 (100 U/mL). After 48 hrs of co-culture, T cells were removed, and organoids were fixed and covered with mounting medium, to be observed using a TCS SPE Leica microscope. Image acquisition was performed by maintaining the same laser power, gain, offset, and magnification (20x). To quantify T cell recruitment and infiltration maximum intensity projections for each analyzed organoid were generated with LAS X Software (Leica). Images of the total PKH26 red fluorescence area present either at the boundary or inside the organoids were analyzed using ImageJ software.

### Live-cell imaging of cytotoxic assays against 3D organoids

A plate containing organoids prepared and treated as above was subjected to image recording by Iris 15 camera (Photometrics) mounted on high content imaging system LIPSI (Nikon Instrument Inc.). Images were taken up to 7 days at identical positions in intervals of 4 hours at 10x magnification. All images were analyzed with NIS Elements software. The percentage of tumor cell lysis was calculated as above.

### Evaluation of anti-MET molecules activity

Target cells (2000/well in 96 well plates) were seeded in complete medium. After 24 hours culture medium was replaced with fresh one containing increasing concentrations of anti-MET small molecules TKIs (JNJ-38877605, Crizotinib, Capmatinib, or PHA665752, all from Selleckchem), or the anti-MET antibody MvDN30 (produced and purified by U-Protein Express BV). Cell viability was determined after further 72 hours by Cell Titer-Glo Luminescent Cell Viability Assay, as described above.

### Determination of MET gene copy number

Genomic DNA from cells was obtained by Maxwell RSC® Cell DNA purification kit (Promega). MET gene copy number was determined by Real-Time qPCR using the Taqman probe Hs04993403_cn (Thermo Fisher Scientific). To normalize gDNA in the samples RNAase-P Taqman probe Hs00468130_cn (Thermo Fisher Scientific) was used. The MET gene copy number was normalized to those of A549 or 293T diploid control cells.

### Analysis of Perforin and Granzyme B secretion

Effector cells were co-cultured in the presence of IL-2 (100 U/mL) with EBC-1 target cells (10^4^ cells/well in a 48 well plate) at a 2:1 ratio. After 48 hours, culture supernatants were collected and analyzed for Perforin and Granzyme B concentrations using an ELISA assay (Diaclone SAS, Besancon, France), as recommended by the manufacturer.

### Analysis of cytokine secretion

Effector cells were co-cultured in the presence of IL-2 (100 U/mL) with EBC-1 target cells (5000 cells/well in a 96 well plate) at a 10:1 ratio. After 48 hours culture supernatants were collected and cytokines quantification was determined with a multi-analyte ELISArray Kit (Human Th1 / Th2 / Th17 Cytokines Multi-Analyte ELISArray Kit, Qiagen).

### In vivo experiments

All procedures in mice were performed according to protocols approved by the Ethical Committee for animal experimentation of the Candiolo Cancer Institute and by the Italian Ministry of Health.

10^6^ EBC-1 cancer cells + 10^6^ T cells (NTD, #948, #949, or Vehicle as control; *n* = 6) were co-injected subcutaneously into the right posterior flank of adult NOD-SCID mice. Tumor size was evaluated every 2 days with a caliper. Tumor volume was calculated as described [39]. Mice were considered tumor positive when the volume was > 30 mm^3^. Animals were euthanized 26 days after cell injection.

Caki-I (3 × 10^6^ cell/mouse) resuspended in 100 μl of Iscove medium + 100 μl of Matrigel matrix (Corning Inc.) were injected subcutaneously into the right posterior flank of adult NOD-SCID mice. After 4 days, animals were randomized into 3 experimental arms (*n* = 6) and assigned to the different treatments: Vehicle (PBS), NTD, and #948 MET-CAR-T. T cells (10^7^/mouse) were delivered by tail vein injection on day 4, 7, 10. Tumor size was evaluated periodically with a caliper. Tumor volume was calculated as above. Mice were euthanized on day 35.

GTR-210 PDXs were generated as described in [40]. Six days after the implant animals were randomized into 3 experimental arms and treated as follows: Vehicle (PBS, *n* = 5), NTD (10^7^ cells/mouse, *n* = 6), and #949 MET-CAR-T (10^7^ cells/mouse, *n* = 7). Tumor size evaluation and tumor volume calculation were done as above. Mice were euthanized on day 56.

### Statistical analysis

Average, standard deviation (SD), and standard error of the mean (SEM) were calculated using Microsoft Office Excel 2010 software (Microsoft Corporation). Statistical significance was determined using One-way Anova or Two-way Anova corrected Bonferroni, using GraphPad Prism software. All the in vitro experiments were repeated at least two times. Figures show one representative experiment.

## Results

### MET-CAR design and MET-CAR-T cell generation

**Fig. 1:**
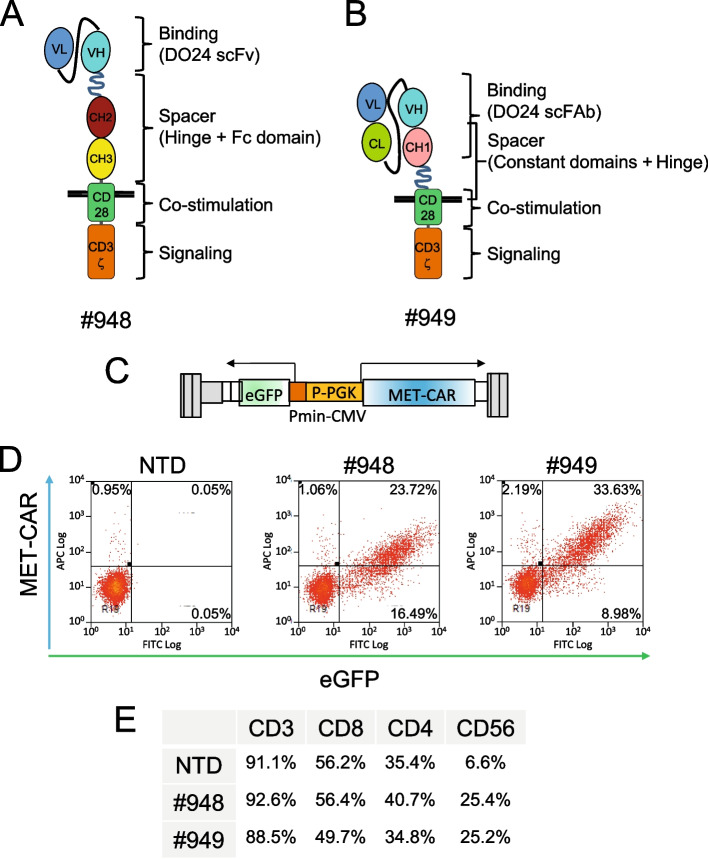
MET-CAR design, expression, and characterization. (**A**, **B**) Schematic drawing of second-generation MET-CARs. #948: MET-CAR with DO24 scFv as binding domain; #949: MET-CAR with DO24 scFAb as binding domain. VL: Variable Light domain; VH: Variable Heavy domain; CL: constant region from human Igκ chain; CH1, CH2, CH3: constant regions 1, 2, 3 from human IgG1 chain; CD28: transmembrane and juxtamembrane regions from human CD28; CD3ζ: zeta region from human CD3. Black lines represent the plasma membrane. (**C**) Schematic drawing of the lentiviral vector (LV) expressing MET-CARs. In grey vector backbone in color the bidirectional expression cassette. P-PGK: human phosphoglycerate kinase promoter; MET-CAR: cDNA encoding for #948 or #949 MET-CAR; Pmin-CMV: minimal promoter from the cytomegalovirus; eGFP: cDNA encoding for the enhanced Green Fluorescent Protein. Pmin-CMV and eGFP are in antisense with respect to the P-PGK and MET-CAR. Arrows indicate the divergent RNA transcripts originating from the bidirectional internal promoter. (**D**) Representative flow-cytometry analysis of MET-CAR and eGFP expression in PBMC transduced with #948-LV or #949-LV. Numbers in the plots indicate the percentage of events for each quadrant. (**E**) Representative flow-cytometry analysis of immune-phenotype markers in PBMC transduced with MET-CAR LVs. The table reports the percentage of expression for each marker in the total cell population. NTD: Not-transduced PBMC

For MET-CAR assembly, a binding domain derived from the DO24 MET antibody [33] was included in a CD28-CD3ζ second-generation CAR. Two different binding domains were generated: i) a single-chain variable fragment (scFv) (Suppl. Fig. 1A); ii) a single-chain chimeric FAb (scFAb) (Suppl. Fig. 1B). After proving that the two single-chain antibody fragments interacted with MET with high affinity (Suppl. Fig. 1C), both the binding sequences were linked in frame to an IgG1-derived spacer, followed by the transmembrane and intracellular regions derived from the T cell receptor complex (Fig. 1A and B). MET-CAR sequences including the scFv (#948 MET-CAR) or the scFAb (#949 MET-CAR) were cloned into a third generation bidirectional lentiviral vector, to coordinately express the CAR and a fluorescent marker (Fig. 1C). Upon transduction of activated human peripheral blood mononuclear cells (PBMC), we obtained a successful membrane expression of both MET-CAR constructs (from 20 to 60%, according to the experiments) (Fig. 1D). Concerning the phenotype of CAR expressing cells, all of the population was CD3^+^, in the majority of the cases co-expressing CD8. A subpopulation was CD56^+^, suggesting potential CAR- and MHC-independent tumor killing properties from this Natural Killer-like cell subset (Fig. 1E).

### MET-CAR-T cytotoxic activity is specific and depends on the level of MET expression

**Fig. 2:**
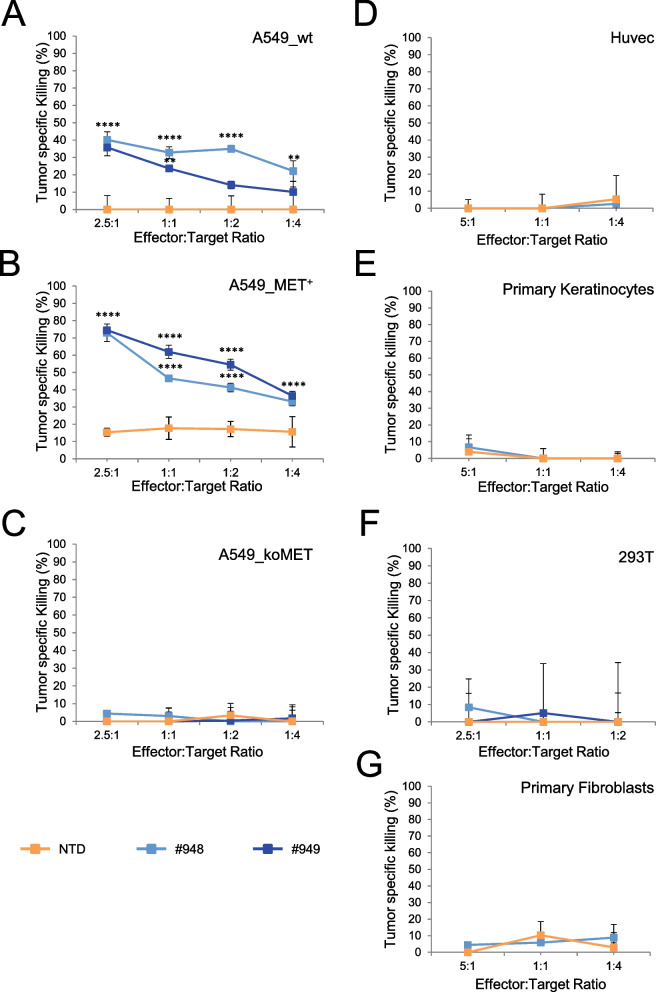
MET-CAR-T killing activity on human cells expressing different levels of the MET receptor at the cell surface. MET-CAR-T specific killing activity at different Effector:Target ratio versus: (**A**) wild type A549 human lung carcinoma cells (A549_wt); (**B**) A549 cells genetically modified to overexpress the MET receptor (A549_MET^+^); (**C**) A549 cells genetically modified to abrogate the expression of the MET receptor (A549_koMET); (**D**) Huvec, human umbilical vein endothelial cells; (**E**) human primary keratinocytes; (**F**) 293 T, not-transformed human kidney epithelial cells; (**G**) human primary fibroblasts. NTD: Not-transduced T cells, #948/#949: MET-CAR-T. Bars: Standard Deviations. Statistical significance between NTD and MET-CAR-T was calculated by Two-way Anova, corrected Bonferroni. Stars indicated *P* values: **, *P* ≤ 0.01; ****, *P* ≤ 0.0001

To assess the killing activity of MET-CAR-T, in vitro viability assays were conducted by incubating T lymphocytes and target cells at different ratios. To define MET-CAR antigen selectivity, A549 human lung carcinoma cells (A549_wt), carrying a wild-type diploid MET gene and expressing a well detectable level of MET at the cell surface in virtually all the cell population (Suppl. Fig. 2A, top panel), were engineered to harbor different levels of MET expression. A higher MET expression compared to the original cell population was obtained upon transduction with MET expressing lentiviral vector particles (A549_MET^+^) (Suppl. Fig. 2A, middle panel). A complete knock-down of MET gene expression was achieved by CRISPR-CAS9 technology, introducing a point mutation disrupting the reading frame of the endogenous MET gene in the cell genome (A549_koMET) (Suppl. Fig. 2A, bottom panel). While MET-CAR-T exerted a modest cytotoxic activity versus A549_wt (Fig. 2A), their effect against A549_MET^+^ cells was remarkable, reaching more than 70% killing at a 2.5:1 effector/target (E:T) ratio (Fig. 2B). The two #948 and #949 MET-CAR-T displayed similar specific killing potency, detectable also at a low E:T ratio (1:4). This activity differed from the low cytotoxic properties exerted in some experiments by not-transduced (NTD) T lymphocytes. Finally, MET-CAR-T tumor-killing activity was completely lost against A549_koMET cells (Fig. 2C), in which MET expression was genetically abrogated. To evaluate the potential risk of ‘on-target/off-tumor’ activity due to tumor unrestricted MET expression, cytotoxicity assays were performed on not-transformed human cells expressing physiological MET levels, such as endothelial Huvec cells, primary epidermal keratinocytes, and embryonic kidney 293 T cells (Suppl. Fig. 2B). MET negative cells, primary skin fibroblasts, were also included in the panel (Suppl. Fig. 2B, bottom panel). While MET-CAR T specifically killed cancer cells expressing MET, the cytotoxic activity against not-transformed cells was absent or negligible (Fig. 2D-G). Thus, the killing properties of MET-CAR-T were fully dependent on the presence of MET receptor on target cells, and the efficacy correlated with the levels of MET expression at the cell surface. Based on a quantitative flow cytometer analysis of surface MET (Suppl. Fig. 3), we found that a threshold of MET expression around 5 folds higher compared to the physiological MET expression detected on normal cells is required to activate a MET-CAR-T effective killing.

### MET-CAR-T cells effectively control MET overexpressing cancer cell growth in vitro and in vivo

**Fig. 3:**
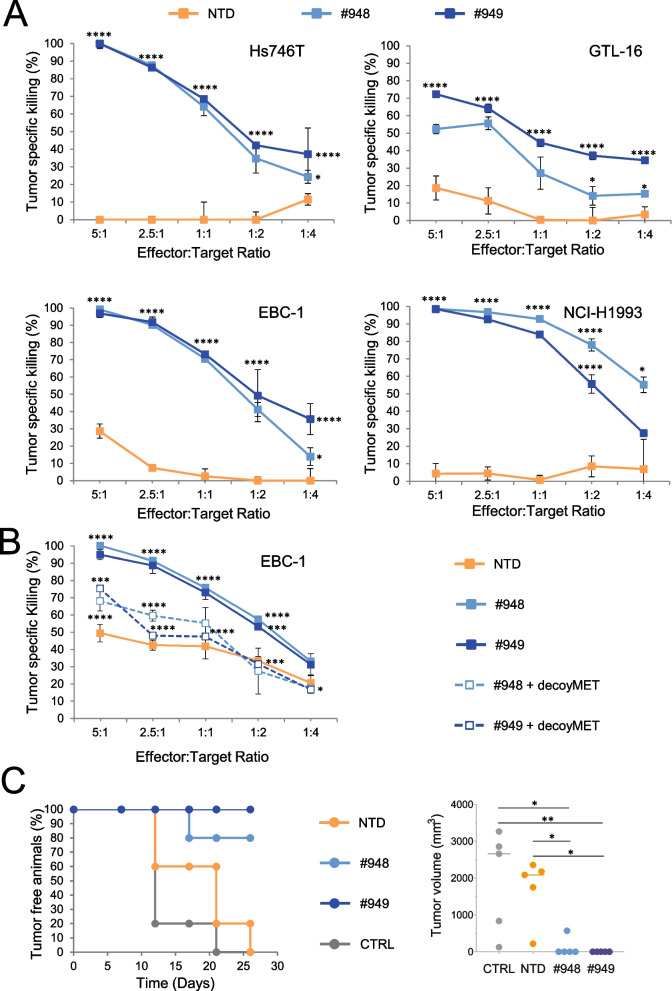
MET-CAR-T cells killing activity on human cancer cells highly overexpressing the MET receptor by mean of MET gene high amplification. (**A**) MET-CAR-T specific killing activity at different Effector:Target ratio versus Hs746T and GTL-16 human gastric carcinoma cells (top panels) or EBC-1 and NCI-H1993 human lung carcinoma cells (bottom panels). (**B**) MET-CAR-T specific killing activity at different Effector:Target ratio versus EBC-1 cells in the presence (dotted lines) or the absence (continuous lines) of soluble MET receptors (decoyMET). (**C**) Left: Tumor onset in NOD-SCID mice injected with EBC-1 and MET-CAR-T cells (ratio 1:1); Right: Tumor volume at sacrifice (26 days after cell injection). NTD: Not-transduced T cells; #948/#949: MET-CAR-T; CTRL: Vehicle (PBS). Bars: Standard Deviations. In panels A and B, statistical significance between NTD and MET-CAR T was calculated by Two-way Anova, corrected Bonferroni. In panel B, the statistical significance between MET-CAR T and MET-CAR-T + decoyMET is also reported. In panel C, statistical significance was calculated by One way Anova. Stars indicated *P* values: *, *P* ≤ 0.05; **, *P* ≤ 0.01; ***, *P* ≤ 0.001; ****, *P* ≤ 0.0001

The above results were confirmed challenging MET-CAR-T against cancer cells featuring extremely high levels of MET receptor on the cell surface, generated by a high grade of MET gene amplification (Suppl. Fig. 4 and Suppl. Table 1). As shown in Fig. 3A, Hs746T and GTL-16 gastric carcinoma cells, EBC-1, and NCI-H1993 non-small cell lung carcinoma cells were powerfully killed by MET-CAR-T. The specificity of MET-CAR-T was assessed by the addition of soluble decoyMET molecules during the cytotoxic assay. DecoyMET, preventing CAR engagement of the MET receptors expressed on the surface of target cells, reduced the killing ability of MET-CAR-T to levels comparable to the NTD effectors (Fig. 3B).

MET-CAR-T were also challenged in an experimental model in vivo. Lung cancer EBC-1 cells were injected subcutaneously in NOD-SCID mice in the presence of MET-CAR-T (E:T ratio = 1:1). This resulted in a remarkable control of tumor growth. At the end of the experiment (26 days after tumor injection), 4 out of 5 mice of the #948 treated group and all the mice treated with #949 MET-CAR-T were still tumor-free (Fig. 3C).

In addition to the killing properties, we evaluated perforin and granzyme B secretion by MET-CAR-T and the production of a panel of cytokines (IL-4, IL-5, IL-6, IL-10, IL-12, IL-13, IL-17a, IFN-γ, TNF-α, G-CSF and TGF-β1), either at baseline or after MET antigen engagement. These analyses are representative of T cell activation and inflammatory responses. To this aim, cell supernatants collected from CAR-T or NTD cells co-cultured with target cells were subjected to ELISA assays. MET-CAR-T increased the secretion of granules containing perforin and granzyme B only in the presence of MET overexpressing target cells (Suppl. Fig. 5). For what concerned cytokine determination in the absence of target cells, NTD-T cells did not produce detectable levels of any of the tested cytokines, and MET-CAR-T produced IFN-γ and IL-13 at very low levels. After MET engagement, MET-CAR-T significantly increased the production of IFN-γ, IL-13, and TNF-α, while NTD-T cells did not. IL-5 was barely detectable in the MET-CAR-T cells supernatants and its levels were not modified in the presence of the target antigen. The levels of all the other analyzed cytokines were undetectable in all the tested conditions (Suppl. Fig. 6).

### MET-CAR-T cells overcome secondary resistance to anti-MET agents

**Fig. 4:**
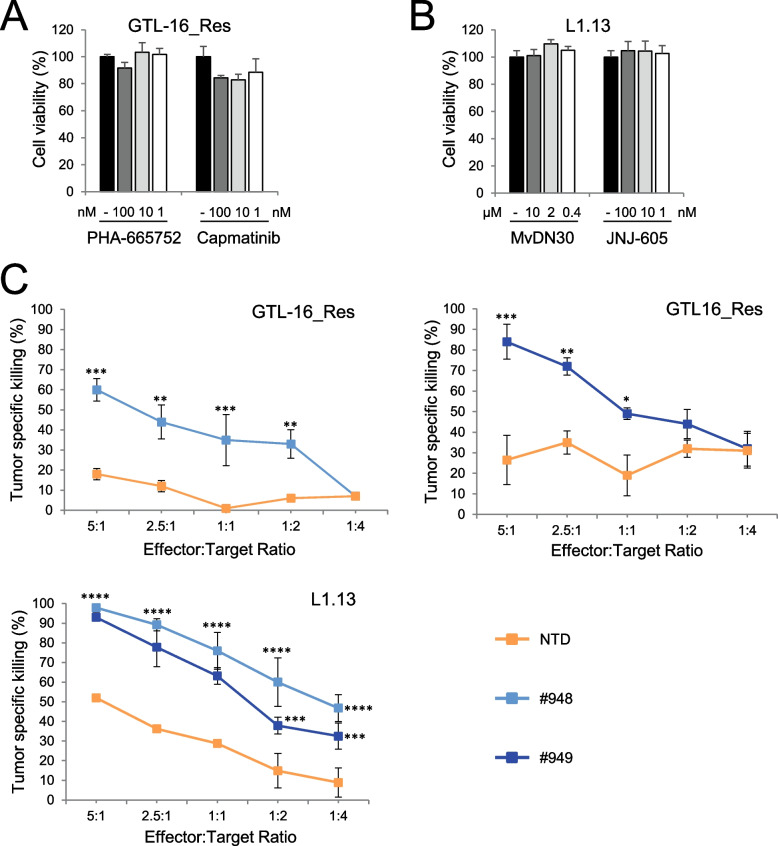
MET-CAR-T killing activity on MET highly amplified human cancer cells resistant to MET targeting agents. Analysis of cell viability after 3 days of treatments with different concentrations of anti-MET agents on human gastric carcinoma cells GTL-16_Res cells (**A**) or Cancer of Unknown Primary L1.13 cells (**B**). PHA-665752, Capmatinib, JNJ-605: small molecule Tyrosine Kinase Inhibitors targeting MET. MvDN30: a MET inhibitory antibody. Graphs report values as percentage of the untreated controls. (**C)** MET-CAR specific killing activity at different Effector:Target ratio versus GTL-16_Res (Top panels) and L1.13 (Bottom panel). NTD: Not-transduced T cells, #948/#949: MET-CAR-T. Bars: Standard Deviations. Statistical significance between NTD and MET-CAR-T was calculated by Two-way Anova, corrected Bonferroni. Stars indicated *P* values: *, *P* ≤ 0.05; **, *P* ≤ 0.01; ***, *P* ≤ 0.001; ****, *P* ≤ 0.0001

The above described tumor models feature a high grade of MET-gene amplification. In this condition, MET represents the driver of the malignancy (*i.e.* cancer cells are MET 'addicted’), thus MET targeting agents are effective [40, 41] and MET-CAR-T may not be considered a priority. Nevertheless, the emergence of clones carrying genetic lesions able to activate MET downstream intracellular transducers can sustain acquired resistance to targeting agents [42]. In this case, an alternative therapeutic approach is needed. Thus, we explored the efficacy of MET-CAR-T against MET amplified cancer cells resistant to anti-MET molecules. One model is represented by GTL-16_Res, generated by prolonged in vitro treatment of the GTL-16 gastric cancer cell line with the anti-MET Tyrosine Kinase Inhibitor (TKI) PHA-665752 [28]. GTL-16_Res cells are characterized by hyperamplification of K-RAS and MET genes, retain MET overexpression (Suppl. Fig. 7A), and are insensible to several MET TKIs (Fig. 4A) and [43]. A second model is represented by L1.13 primary cells, derived from a metastatic lesion of a Cancer of Unknown Primary (CUP) patient characterized by the presence of high MET gene amplification (Suppl. Table 1) and MET overexpression (Suppl. Fig. 7B) and [15]. Nevertheless, an activating mutation on the BRAF gene turns off METactivation, rendering L1.13 cells unresponsive to anti-MET agents, either a specific small molecule TKI (JNJ-605) or a blocking antibody (MvDN30) (Fig. 4B). Importantly, MET-CAR-T exerted a potent cytotoxic effect against cancer cells, efficiently breaking tumor resistance in both models (Fig. 4C).

### MET-CAR-T cells display high cytotoxic activity against MET overexpressing cancer cells, organoids, and tumor xenografts unresponsive to MET targeting molecules

**Fig. 5:**
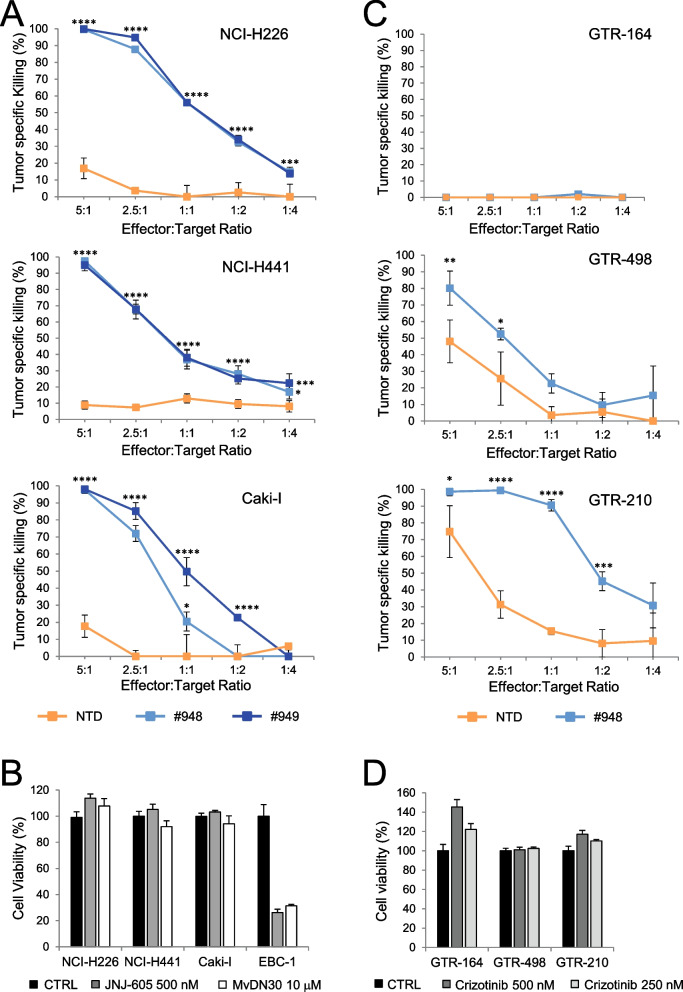
MET-CAR-T killing activity on human cancer cells overexpressing the MET receptor. (**A**) MET-CAR-T specific killing activity at different Effector:Target ratio versus NCI-H226 and NCI-H441 human lung carcinoma cells, and Caki-1 human renal carcinoma cells. (**B**) Analysis of cell viability after 3 days of treatments with a MET-specific small molecule TKI (JNJ-605) or with a MET-blocking antibody (MvDN30) on NCI-H226, NCI-H441, and Caki-1 cells. Graph reports values as percentage of the untreated controls (CTRL). MET 'addicted' EBC-1 cells has been included in the assay as positive control. (**C**) MET-CAR-T specific killing activity at different Effector:Target ratio versus primary gastric carcinoma cells expressing different levels of MET receptor at the cell surface. GTR-164: MET negative cells; GTR-468 and GTR-210 MET overexpressing cells. (**D**) Analysis of cell viability after 3 days of treatments with different concentrations of a MET targeting small molecule TKI (Crizotinib). Graph reports values as percentage of the untreated controls. NTD: Not-transduced T cells, #948/#949: MET-CAR-T. Bars: Standard Deviations. Statistical significance was calculated by Two-way Anova, corrected Bonferroni. Stars indicated *P* values: *, *P* ≤ 0.05; **, *P* ≤ 0.01; ***, *P* ≤ 0.001; ****, *P* ≤ 0.0001

**Fig. 6:**
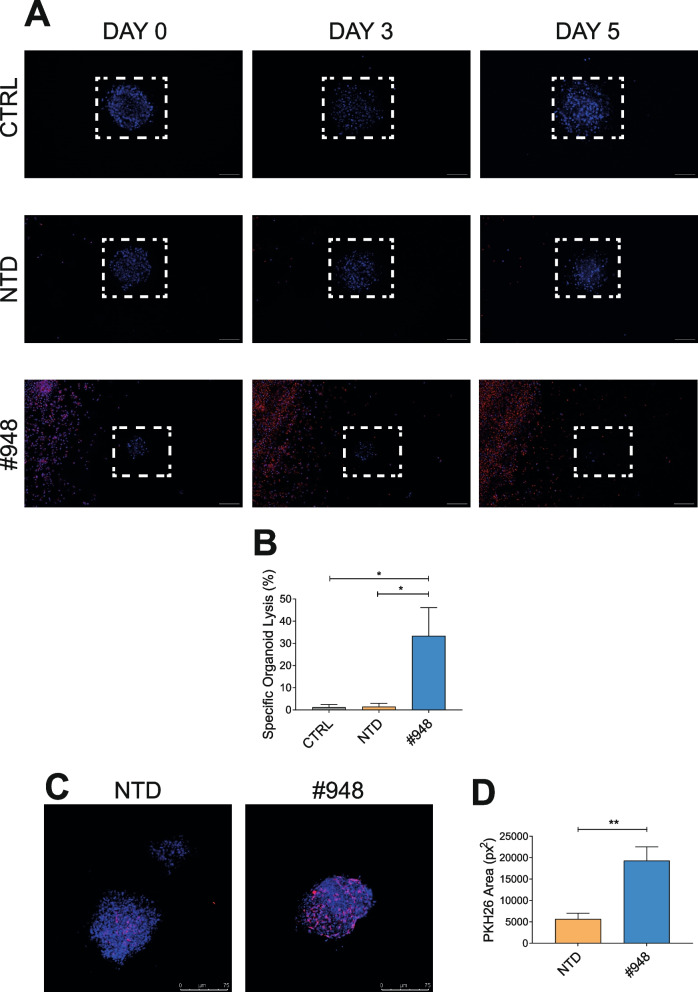
MET-CAR-T killing activity on tumor organoids derived from primary human gastric cancer cells overexpressing the MET receptor. (**A**) Representative pictures of organoids derived from GTR-468 primary gastric carcinoma cells grown in a three-dimensional matrix in the presence of #948 MET-CAR-T for 3 or 5 days. Cancer cells were stained with NucBlue (Blue signal), and T cells were stained with PHK26 (Red signal) Magnification, 20x; scale bars, 75 μm. (**B**) Tumor organoid elimination mediated by MET-CAR-T quantified by measuring NucBlue fluorescence loss after 5 days of co-culture. (**C**) Representative maximum intensity projections of confocal microscopy images of GTR-468-derived organoids (blue) treated with MET-CAR-T or not-transduced T cells (red; E:T ratio, 2:1). Confocal microscopy images taken after 48 hours of co-culture. Magnification, 20x; scale bars, 75 μm. (**D**) Quantification of MET-CAR-T infiltration into GTR-468-derived organoids by the analysis of red fluorescence PKH26 area (px^2^). CTRL: Untreated organoids; NTD: Organoids treated with not-transduced T cells; #948: Organoids treated with MET-CAR-T. Bars: SEM. Statistical significance was calculated by Two-way Anova, corrected Bonferroni. Stars indicated *P* values: * *P* ≤ 0.05; ** *P* ≤ 0.01

**Fig. 7:**
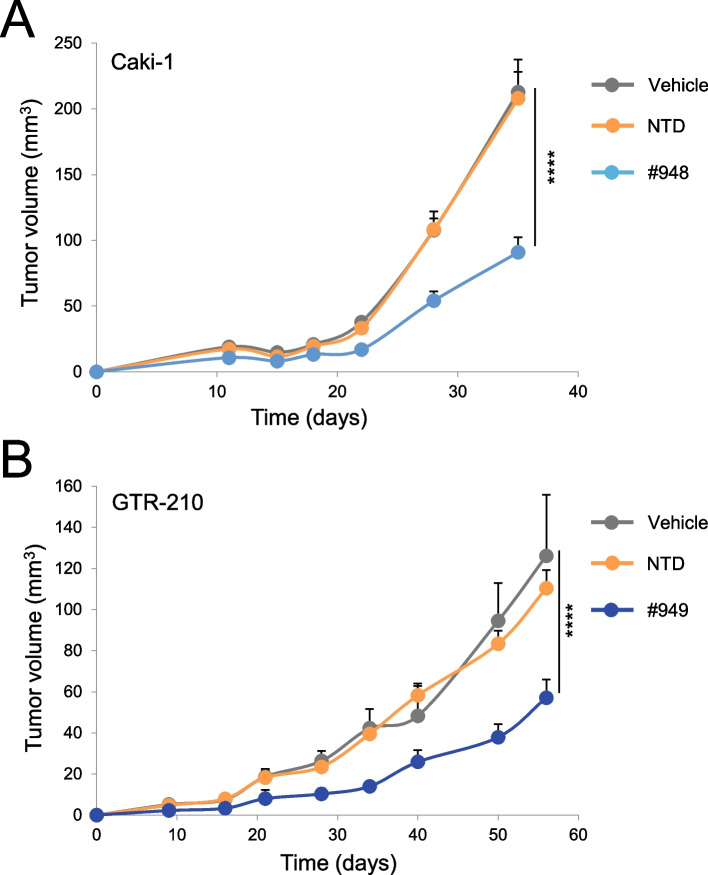
MET-CAR-T therapeutic activity in experimental tumors overexpressing the MET receptor. (**A**) Caki-1 tumor growth in NOD-SCID mice treated with 10^7^ MET-CAR-T by intravenous injection on days 4, 7, and 11. (**B**) GTR-210 tumor growth in NOD-SCID mice treated with 10^7^ MET-CAR-T by intravenous injection on day 6. Vehicle: PBS; NTD: Not-transduced T cells; #948/#949: MET-CAR-T. Bars: Standard Error of the Mean. Statistical significance between Vehicle and MET-CAR-T was calculated by Two-way Anova, corrected Bonferroni. Stars indicated *P* values: ****, *P* ≤ 0.0001

Another condition in which MET targeting molecules are not effective in inhibiting the growth of cancer cells displaying MET constitutive activation is when MET overexpression is sustained by low copy number gain or by transcriptional upregulation of the diploid gene. Thus, we selected cancer cell lines of different origin (NCI-H226, NCI-H441 non-small cell lung carcinoma, and Caki-I clear cell renal carcinoma) characterized by MET overexpression in the absence of high MET gene amplification (Suppl. Fig. 8A and Suppl. Table 1) and challenged them with MET-CAR-T or with anti-MET molecules (*i.e.* the TKI JNJ-605 or the antibody MvDN30). MET-CAR-T efficiently counteracted cancer cell growth (Fig. 5A), while anti-MET agents did not affect cell viability (Fig. 5B). The analysis was then extended to primary gastric carcinoma cell lines (GTR-164, GTR-498, GTR-210) derived from bioptic material expanded in immunocompromised mice (Patient Derived Xenograft) expressing different MET levels (Suppl. Fig. 8B), in the absence of high MET gene amplification (Suppl. Table 1). Also in these models, MET-CAR-T killing was dependent on cell surface MET expression (Fig. 5C), while targeted therapies were ineffective (Fig. 5D). MET-CAR-T killing properties were also evaluated against tumor organoids. Organoids are in vitro 3D cultures recapitulating in vivo architecture, functions, and genetic signature of their tissue of origin, thus representing a highly reliable tool for in vitro analysis [44]. MET-CAR-T disrupted GTR-498-derived organoids more efficiently than paired NTD-T cells (Fig. 6A, B). In line with these results, we confirmed that MET-CAR-T were able to exploit a higher infiltration of GTR-498-derived organoids compared to paired NTD-T cells (Fig. 6C, D, Suppl. Videos S1, S2). Finally, we tested the efficacy of MET-CAR-T in vivo, against MET overexpressing tumors generated by subcutaneous injection of Caki-1 cells or by implanting the GTR-210 PDX. MET-CAR-T were delivered by intravenous injection and tumor growth was monitored periodically. In both models, MET-CAR-T significantly delayed tumor growth, while NTD-T cells were ineffective. At the end of the experiment, tumor masses were on average 57.3% (Caki-1, Fig. 7A) and 54.7% (GTR-210, Fig. 7B) reduced compared to animals treated with vehicle.

## Discussion

In this paper, we describe a novel precision medicine strategy based on CAR-mediated redirection of the immune response against MET overexpressing cancers not eligible for treatments with MET targeting molecules. We demonstrated that MET-CAR-T successfully controlled tumor expansion in the presence of MET overexpression, sparing not transformed cells expressing MET at physiological levels. This activity was independent of the role of MET in sustaining the disease (*i.e.* if MET was the gene driving the malignancy or not). Moreover, MET-CAR-T killing was effective also when overexpressed MET was not phosphorylated (as in the case of L1.13 CUP cells) or when the activation of downstream transducers bypassed MET signaling impairment (as in the case of GTL-16_Res). MET-CAR-T activity proved to be specific, as it was always much more potent compared to T cells not expressing MET-CAR, even when those effectors induced detectable levels of cytotoxicity. This background, scored in some experiments, was probably related to the presence of CD56^+^ immune cells that expanded under the IL-2 enriched culture conditions.

MET overexpression is quite frequent in solid tumors. In the majority of cases, overexpression is sustained by transcriptional upregulation of the MET gene, whose promoter includes sequences interacting with stress-responsive transcriptional factors [45, 46]. Cancer cells exploit MET activation induced by receptor overexpression to sustain an adaptive response to adverse micro-environmental conditions [21]. Thus, MET signaling represents a useful tool for tumor endurance but is not critical because other genes are driving the malignancy. In this contest, MET targeting agents will not exert a major therapeutic response, being the tumor primary resistant to this kind of therapy. On the contrary, the simple presence of high receptor levels on the cancer cell surface renders them a suitable target for MET-CAR-T. In this respect, MET-CAR immunotherapy holds potential for a broad and successful application.

When MET overexpression is sustained by a high grade of gene amplification (2–4% of total cancers), MET can be the ‘driver’ of the malignancy. In this condition, MET targeting molecules are predicted to be effective, and therefore they should be the elective treatment choice. Nevertheless, clinical experience indicates that the emergence of resistant clones eventually limits the response duration. Secondary resistance is indeed the major drawback of targeted therapies, especially when the inhibition of receptor signaling is obtained by TKIs. Nowadays, several approaches to address the issue of secondary resistance are under evaluation. To be successful, they require a rational driven application, based on the knowledge of the mechanism(s) sustaining resistance. As cancer cells can develop different mechanisms of secondary resistance, an effective solution addressing only one of them will not permanently solve the problem. When secondary resistance is sustained by an identified newly emerged driver mutation, the use of combination therapies able to concomitantly hit more than one oncogenic pathway could be possible. Nevertheless, it results to be often cumbersome, potentially prone to toxicity, and predicted as well to be not durable, because tertiary resistance is frequently observed. Considering all these issues, MET-CAR-T immunotherapy can represent a valuable therapeutic opportunity to be explored in cases of secondary resistance. Indeed, the major requirement for its application is the high level of MET expression, a feature characterizing the majority of MET 'addicted' malignancies, which is bypassed - but not abolished - during resistance.

CAR-T cells targeting MET have been previously developed, showing the potential of this therapeutic strategy in preclinical models, either alone or in combination with chemotherapy or targeting agents. These studies focused on sub-histotypes of the breast [47], lung [48], liver [49], and kidney tumors [50], on gastric carcinomas [51], and mesotheliomas [52]. They provided evidence of MET-CAR-T specificity by comparing models expressing or not MET, without analyzing possible differences in killing activity related to the level of MET expression. The MET-CAR constructs here described induced efficient killing only in the presence of MET overexpression, being able to discriminate between cancer cells and normal tissues, the latter usually expressing a significantly lower level of the receptor. Notably, HER-2 and EGFR -CAR-T activation only in the presence of a high surface density of their relative target has been described and has been indicated as a useful safety feature [53–55]. This property could depend on the CAR design, because the binding domain (its affinity, avidity, and accessibility) as well as the spacer (its dimension, composition, and flexibility) included in the CAR extracellular region, can significantly diversify the cytotoxic response [56, 57]. As the involvement of different modules crucially and unpredictably influences the effector functions, choosing the right CAR construct is an essential but difficult task. In this perspective, the availability of differently designed MET-CARs could significantly improve the chances of obtaining the desired therapeutic outcome.

Recently, two different MET targeting drug-conjugated antibodies (ADC) – Telisotuzumab-Vedotin/ABBV-399 and METxMET-M114 [58, 59] - have been proposed for an application similar to the MET-CAR immunotherapy here discussed. Through the antibody, a cytotoxic compound is shuttled to the tumor site, where it carries out its killing properties, provided that the tumor overexpresses MET. In theory, redirection of the immune response by CAR engineering could be considered safer compared to ADCs, as genetically modified effectors are working within a physiological network still subjected to the governance of different endogenous factors, while ADCs control is elusive, relying only on a careful evaluation of the dose that can be delivered without inducing toxicity. Further studies are needed to clarify which, of the two strategies, will give rise to the best therapeutic response avoiding - as much as possible - unwanted side effects.

As a general perception, the use of CAR-T is considered potentially risky, because severe side effects, such as cytokine release syndrome or neurological toxicities, have been observed in some of the treated patients [60]. Our in vitro data showed that the pattern of cytokines released by MET-CAR-T cells is not dramatically modified, neither by CAR expression nor upon target engagement. This is a preliminary positive indication about a possible good level of safety of the MET-CAR-T therapy. In addition, mice receiving MET-CAR-T did not show signs of pain and, upon gross autopsy, organ toxicity has not been observed (data not shown). In future studies, it will be important to define both the lowest number and the minimal frequency of the infused effector cells required to obtain the therapeutic outcome, since this represents a way to limit the extent of side effects. In addition, the replacement of the cDNA expressing eGFP with an inducible suicide gene in the lentiviral construct will be considered. This allows ‘on-demand’ control of the effector viability, improving considerably the safety of the therapy [61, 62].

MET-CAR immune therapy inevitably focuses on solid tumors. While CAR-cell adoptive transfer showed dramatic potency for the treatment of B-cell malignancy, its applications in solid tumors have not yet achieved striking results [63]. In this setting, a relevant matter to be addressed is the lack of specific cancer targets, which renders challenging the elimination of tumor cells without hitting healthy tissues. At present, the best option available for solid tumor treatment by CAR immunotherapy is probably the identification of a tumor-associated antigen (*i.e.* a target overexpressed in cancer cells but also found in normal cells at lower levels) in place of a tumor-specific one (found on tumor cells and not expressed or virtually absent in normal cells). Considering this option, a MET-CAR should be an appropriate choice. As discussed above, MET overexpression can be considered a hallmark of transformation, due to its primary role during cancer onset and progression. Moreover, being MET expression an inherent distinctive trait of several cancer stem cell types [3, 64], MET-CAR effectors could potentially hit the inner roots of the malignancy. For what concerns MET expression in normal tissue, this is absent or low, because the physiological role of MET during adult life is essentially limited to organ regeneration [19]. Thus, even if MET expression is not completely restricted to tumor cells, the delta in expression between normal versus transformed tissues can keep under control the ‘on-target/off-tumor’ activity. Previous works suggested addressing the ‘on-target/off-tumor’ toxicity delivering MET-CAR expressing cells intra-tumor [47, 51] or in anatomically delimited spaces [52]. Nevertheless, this regional delivery does not completely guarantee the absence of effector diffusion through the body [47] and considerably limits the feasibility of the therapy.

Another relevant issue that hampers the efficacy of CAR cell therapy in solid tumors is the presence of intrinsic intracellular pathways sustaining immune tolerance. In the case of MET-CAR, different strategies have been developed aimed at addressing this point. They included the construction of a MET/PDL-1 tandem CAR blocking the interaction between PD-1 and PD-L1 [65], the development of a dual-function MET/PD-1 CAR suitable to prevent CAR-T exhaustion [66], and the generation of a MET-CAR incorporating a PD-1/CD28 chimeric-switch receptor, boosting the T cell activity by reverting the PD-1 inhibitory signaling [67]. Future studies are required to define if the above-listed molecular engineering strategies are better than using MET-CAR cells in combination with immune checkpoint targeting agents.

A highly efficient MET-CAR strategy having the potential to break solid tumor barrier must also incorporate features suitable to maximize the effector’s ability to infiltrate the malignant lesions, and to overcome the unfavorable microenvironment characterized by high concentrations of immunosuppressive molecules and cells. Thus, more complex engineering strategies of the MET-CAR expressing immune effectors suitable for a tumor-site restricted delivery of enzymes able to degrade the extracellular matrix [68] and/or of favorable cytokines [69, 70] could be considered a highly sought step behind.

Finally, the selection of MET negative transformed cells or expressing MET at a low level is an event that can occur under the therapy pressure, as a consequence of tumor heterogeneity and adaptability. In this perspective, the option of expressing the MET-CAR in effector cells characterized by intrinsic CAR independent killing properties must be evaluated, to efficiently counteract clonal selection events [71].

## Conclusions

We designed and validated at the pre-clinical level an immunotherapy strategy against MET overexpressing cancer cells. This potentially offers a treatment option for cancers in which MET receptor is present at high levels on the cell surface but they are not eligible for anti-MET targeting molecules, due to primary or secondary resistance of the malignant lesion.

## Supplementary Information

**Additional file 1. Supplementary Video 1.** NTD-T cell infiltration of GTR-498-derived 3D organoids.

**Additional file 2. Supplementary Video 2.** MET-CAR-T cell infiltration of GTR-498-derived 3D organoids.

**Additional file 3: Supplement Table 1.** MET gene amplification in different cell lines and primary cells derived from human tumors of different origin.

**Additional file 4: Supplementary Figure 1.** Design and binding properties of DO24 single chain antibody fragments. **Supplementary Figure 2.** Analysis by flow cytometry of cell surface MET expression. **Supplementary Figure 3.** Quantitative flow cytometer analysis of surface MET levels in A549 wild type, genetically modified, and not transformed human cells. **Supplementary Figure 4.** Analysis by flow cytometry of cell surface MET expression in carcinoma cells featuring MET overexpression due to high MET gene copy number. **Supplementary Figure 5.** Analysis of perforin and granzyme B concentrations in the culture supernatants of T cells co-cultured with target cells expressing different surface MET levels. **Supplementary Figure 6.** Analysis of cytokine expression in the culture supernatants of T cells expressing the MET-CAR co-cultured or not with target cells. **Supplementary Figure 7.** MET expression on carcinoma cells featuring MET gene amplification and overexpression resistant to anti-MET agents. **Supplementary Figure 8.** MET expression analyzed on carcinoma cells featuring MET overexpression due to transcriptional upregulation of the MET gene, present as diploid or at a low copy number.

## Data Availability

All data related to this study are included in this paper and its supplementary information files. No data sets were generated or analyzed during the current study. Experimental details are available from the corresponding author on reasonable request.

## Abbreviations

ABC: Antibody Binding Capacity
ADC: Antibody Drug Conjugated
CAR: Chimeric Antigen Receptor
CUP: Cancer of Unknown Primary
EGFR: Epidermal Growth Factor Receptor
eGFP: Enhanced Green Fluorescent Protein
G-CSF: Granulocyte Colony Stimulating Factor
Ig: Immunoglobulin
IL: Interleukin
INF: Interferon
LV: Lentiviral Vector
PBMC: Peripheral Blood Mononuclear Cells
ScFAb: Single chain FAb
ScFv: single chain variable Fragment
TGF: Transforming Growth Factor
TKI: Tyrosine Kinase Inhibitor
TNF: Tumor Necrosis Factor
