# Supplementary material for: Efficacy of CAR-T immunotherapy in MET overexpressing tumors not eligible for anti-MET targeted therapy
Source: J Exp Clin Cancer Res. 2022 Oct 21;41:309. doi: 10.1186/s13046-022-02479-y (PMC9585715; doi:10.1186/s13046-022-02479-y)
Supplement: Supplementary file 3 — Additional file 3: Supplement Table 1. MET gene amplification in different cell lines and primary cells derived from human tumors of different origin. [file 13046_2022_2479_MOESM3_ESM.pdf]

MET gene amplification in different cell lines and primary cells derived from human tumors of different origin

Cell line	Tumor origin	Fold Amplification
A549	Lung	1# ± 0.06
EBC-1	Lung	11.9 *
GTL-16	Stomach	10.42# ± 0.69
Hs746T	Stomach	17.9*
NCI-H1993	Lung	7.8*
NCI-H226	Lung	1.13# ± 0.07
NCI-H441	Lung	2.37# ± 0.15
Caki-I	Kidney	2.14# ± 0.04
GTR-164	Stomach	0.96^ ± 0.13
GTR-498	Stomach	1.4^ ± 0.12
GTR-210	Stomach	1.13^ ± 0.41
L1.13	CUP	19.8§
GTL-16_Res	Stomach	2-3 folds higher than GTL-16@

Fold change in MET gene copy number with respect to the diploid cell line A549, determined by real-time qPCR.

^ Fold change in MET gene copy number with respect to the diploid cell line 293T, determined by real-time qPCR.

*Data from Pennacchietti et al., Can Res 2014; PMID: 25217525

§ Data from Virzì et al. PNAS 2016; PMID: 30224486

@Data from Cepero et al., Cancer Res 2010; PMID: 20841479
