# Supplementary material for: Efficacy of CAR-T immunotherapy in MET overexpressing tumors not eligible for anti-MET targeted therapy
Source: J Exp Clin Cancer Res. 2022 Oct 21;41:309. doi: 10.1186/s13046-022-02479-y (PMC9585715; doi:10.1186/s13046-022-02479-y)
Supplement: Supplementary file 4 — Additional file 4: Supplementary Figure 1. Design and binding properties of DO24 single chain antibody fragments. Supplementary Figure 2. Analysis by flow cytometry of cell surface MET expression. Supplementary Figure 3. Quantitative flow cytometer analysis of surface MET levels in A549 wild type, genetically modified, and not transformed human cells. Supplementary Figure 4. Analysis by flow cytometry of cell surface MET expression in carcinoma cells featuring MET overexpression due to high MET gene copy number. Supplementary Figure 5. Analysis of perforin and granzyme B concentrations in the culture supernatants of T cells co-cultured with target cells expressing different surface MET levels. Supplementary Figure 6. Analysis of cytokine expression in the culture supernatants of T cells expressing the MET-CAR co-cultured or not with target cells. Supplementary Figure 7. MET expression on carcinoma cells featuring MET gene amplification and overexpression resistant to anti-MET agents. Supplementary Figure 8. MET expression analyzed on carcinoma cells featuring MET overexpression due to transcriptional upregulation of the MET gene, present as diploid or at a low copy number. [file 13046_2022_2479_MOESM4_ESM.pdf]

A

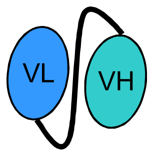

DO24 scFv

METDTILLWVLLLWVPGSTGDIELTQSPSYLAASPGETITINCRASKSISKYLAWYQEKPGKT
NKLLIYSGSTLQSGIPSRFSGSVSGTDFTLTISSLEPEDFAMYYCQQHNEYPFTFGSGTKLEI
KRGGGGSGGGGSGGGGSGGGGSGGGGSEQVKLQESGPGLVAPSQSLSITCAVSGFSLTDY
TIHWIRQPPGKGLEWLGVIWSGGSTNYNSALMSRLNIHKDNSKSQVFLEMNSLQTDDTAIYY
CASPYYRYDGPFAYWGQGTLVTVSATGTRGAAWSHPQFEKGAAWSHPQFEKGAAWSHPQ
FEKGAA*HHHHHH*

B

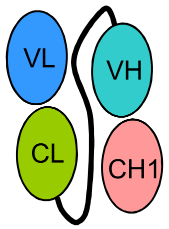

DO24 scFAb

METDTILLWVLLLWVPGSTGDIELTQSPSYLAASPGETITINCRASKSISKYLAWYQEKPGKT
NKLLIYSGSTLQSGIPSRFSGSVSGTDFTLTISSLEPEDFAMYYCQQHNEYPFTFGSGTKLEI
KRTVAAPSVFIFPPSDEQLKSGTASVVCLLNNFYPREAKVQWKVDNALQSGNSQESVTEQD
SKDSTYSLSSTLTLSKADYEKHKVYACEVTHQGLSSPVTKSFNRGECGGSSGSGSGSTGTS
SSGTGTSAGTTGTSASTSGSGSGGGGGSGGGGSAGGTATAGASSGSQVKLQESGPGLVAP
SQSLSITCAVSGFSLTDYTIHWIRQPPGKGLEWLGVIWSGGSTNYNSALMSRLNIHKDNSKS
QVFLEMNSLQTDDTAIYYCASPYYRYDGPFAYWGQGTLVTVSAASTKGPSVFPLAPSSKST
SGGTAALGCLVKDYFPEPVTVSWNSGALTSGVHTFPAVLQSSGLYSLSSVVTVPSSSLGTQ
TYICNVNHKPSNTKVDKKVEPKSCDKTHTTRGAAWSHPQFEKGAAWSHPQFEKGAAWSHP
QFEKGAA*HHHHHH*

C

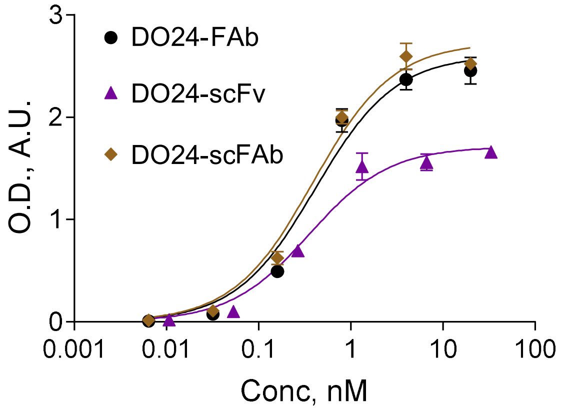

Antibody	Kd (nM)	Bmax (A.U.)
DO24-FAb	0.413 ± 0.089	2.607 ± 0.119
DO24-scFv	0.355 ± 0.069	1.714 ± 0.066
DO24-scFAb	0.391 ± 0.065	2.731 ± 0.095

Supplementary Figure 1. Design and binding properties of DO24 single chain antibody fragments. (A) Schematic drawing and amino acid sequence of DO24 scFv (single chain variable fragment). (B) Schematic drawing and amino acid sequence of DO24 scFAb (single chain Fab). VL (Blue): Variable Light domain; VH (Tiffany): Variable Heavy domain; CL (Green): constant region from human Igκ chain; CH1 (Pink): constant region 1 from human IgG1 chain. Orange: signal peptide; Black: linker; Grey: Strep and Histidine Tags. (C) ELISA binding assay. Extracellular domain of MET was in solid phase, and increasing concentration of DO24 derivatives were in liquid phase. As reference DO24 Fab was included in the assay. On the right, affinity constant (Kd) and maximal binding (Bmax) of DO24 derivatives are reported. OD: Optical density; AU: arbitrary units.

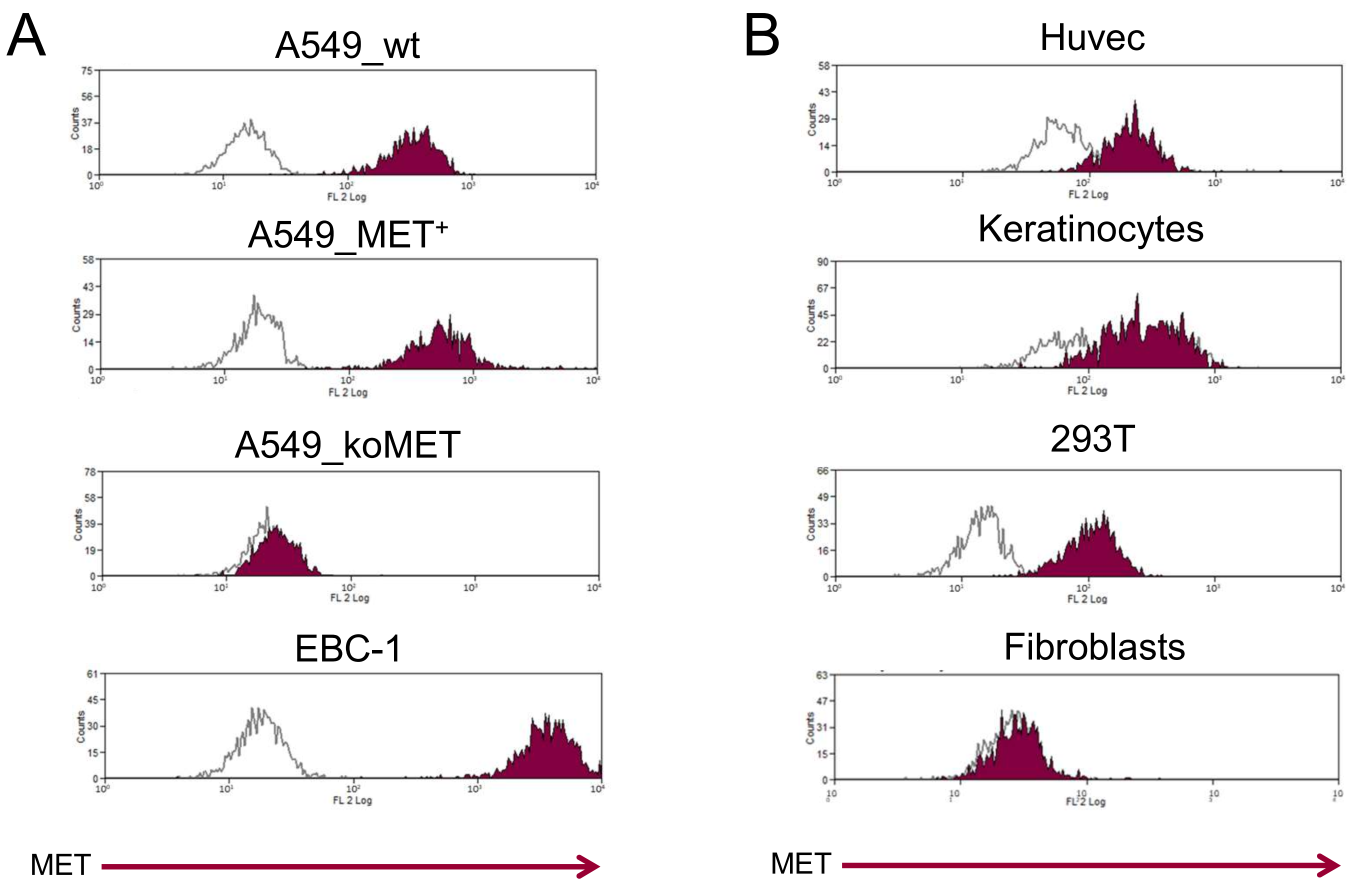

Supplementary Figure 2. Analysis by flow cytometry of cell surface MET expression. (A) A549 human lung carcinoma cells expressing different levels of MET on the cell surface by genetic engineering. A549_wt: A549 carrying wt MET gene; A549_MET^{+}: A549 genetically modified to express higher MET levels; A549_koMET: A549 not expressing MET; EBC-1: human lung carcinoma cells highly overexpressing MET included in the panel as positive control. (B) Not transformed human cells. Huvec: human umbilical vein endothelial cells; Keratinocytes: human primary epidermal keratinocytes; 293T: human embryonic kidney epithelial cells; Fibroblats: human primary skin fibroblasts. Isotype controls for each cell line are shown in grey. Counts: number of the detected events. Surface MET expression levels for each cell lines have been quantified and are reported in Suppl. Figure 3.

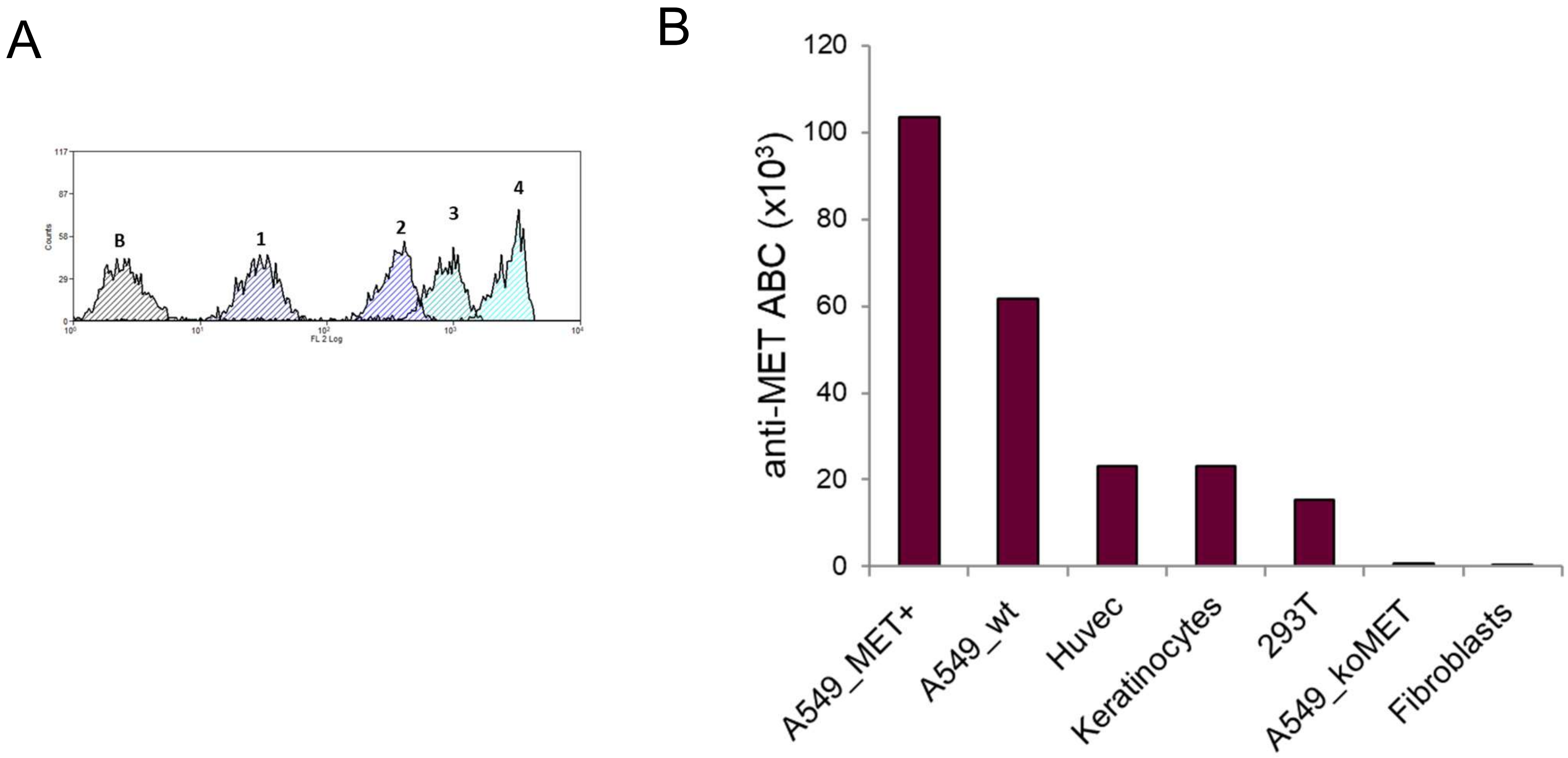

Supplementary Figure 3. Quantitative flow cytometer analysis of surface MET levels in A549 wild type, genetically modified, and not-transformed human cells. (A) Flow-cytometry histogram exhibiting the different 5 populations of microbeads used to built the calibration curve. Microbeads were stained with saturating amount of the anti-MET antibody used to label the cells. Peak B: microbeads not loaded with the antibody, set to fall into the first decade; Peaks 1-4: microbeads with increasing levels of Fc-specific capture antibody. (B) Graph showing the Antibody Binding Capacity (ABC, number of antibodies bound per cell) for each cell population analyzed. ABC has been calculated using the median values obtained in the analysis shown in Suppl. Fig.2, subtracted of the median value of each relative isotype control.

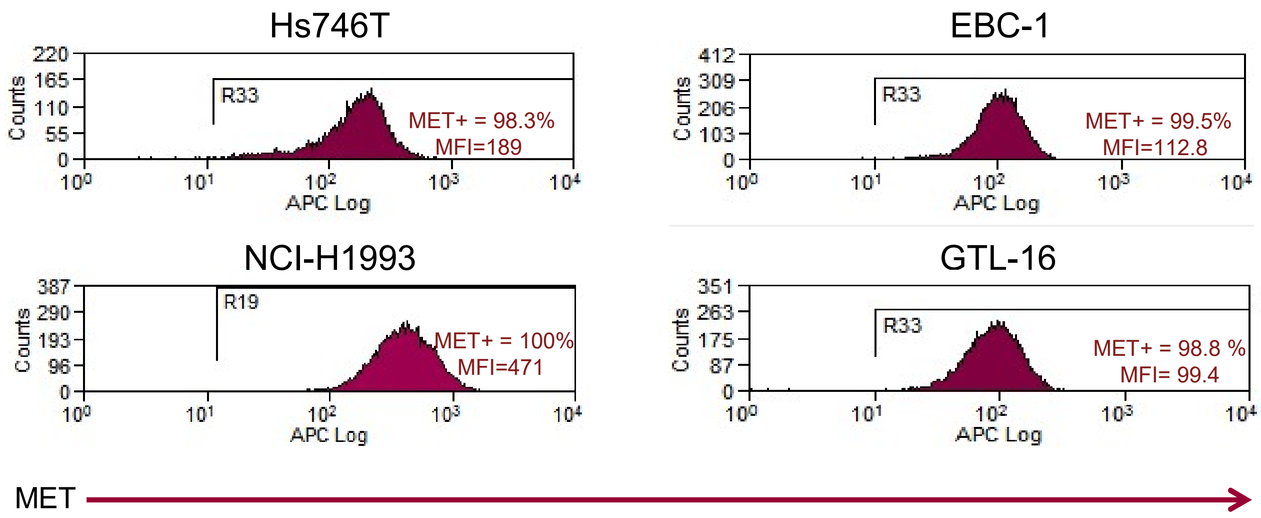

Supplementary Figure 4. Analysis by flow cytometry of cell surface MET expression in carcinoma cells featuring MET overexpression due to high MET gene copy number. Percentage of MET positive cells and Mean Fluorescence Intensity (MFI) are indicated in the plots. Counts: number of the detected events.

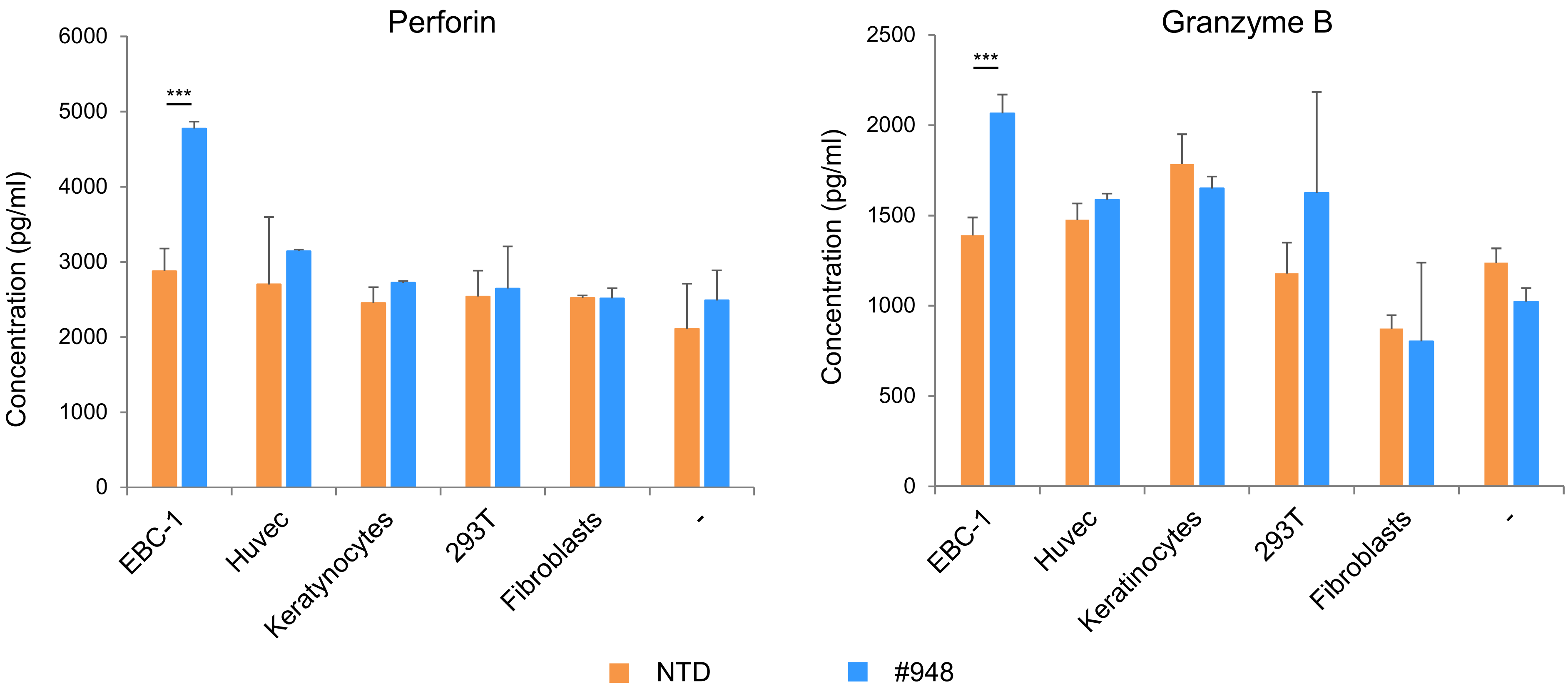

Supplementary Figure 5. Analysis of perforin (Left) and granzyme B (Right) concentrations in the culture supernatants of T-cells co-cultured for 48 hrs with target cells expressing different surface MET levels (Effector:Target ratio = 2:1). EBC-1: cancer cells expressing high levels of surface MET; Huvec, Keratynocytes, 293T: not-transformed cells expressing physiological MET levels; Fibroblasts: MET-negative not transformed cells. NTD: Not transduced T cells; #948: MET-CAR T cells. Bars: Standard Deviations. ***: $P \leq 0.001$.

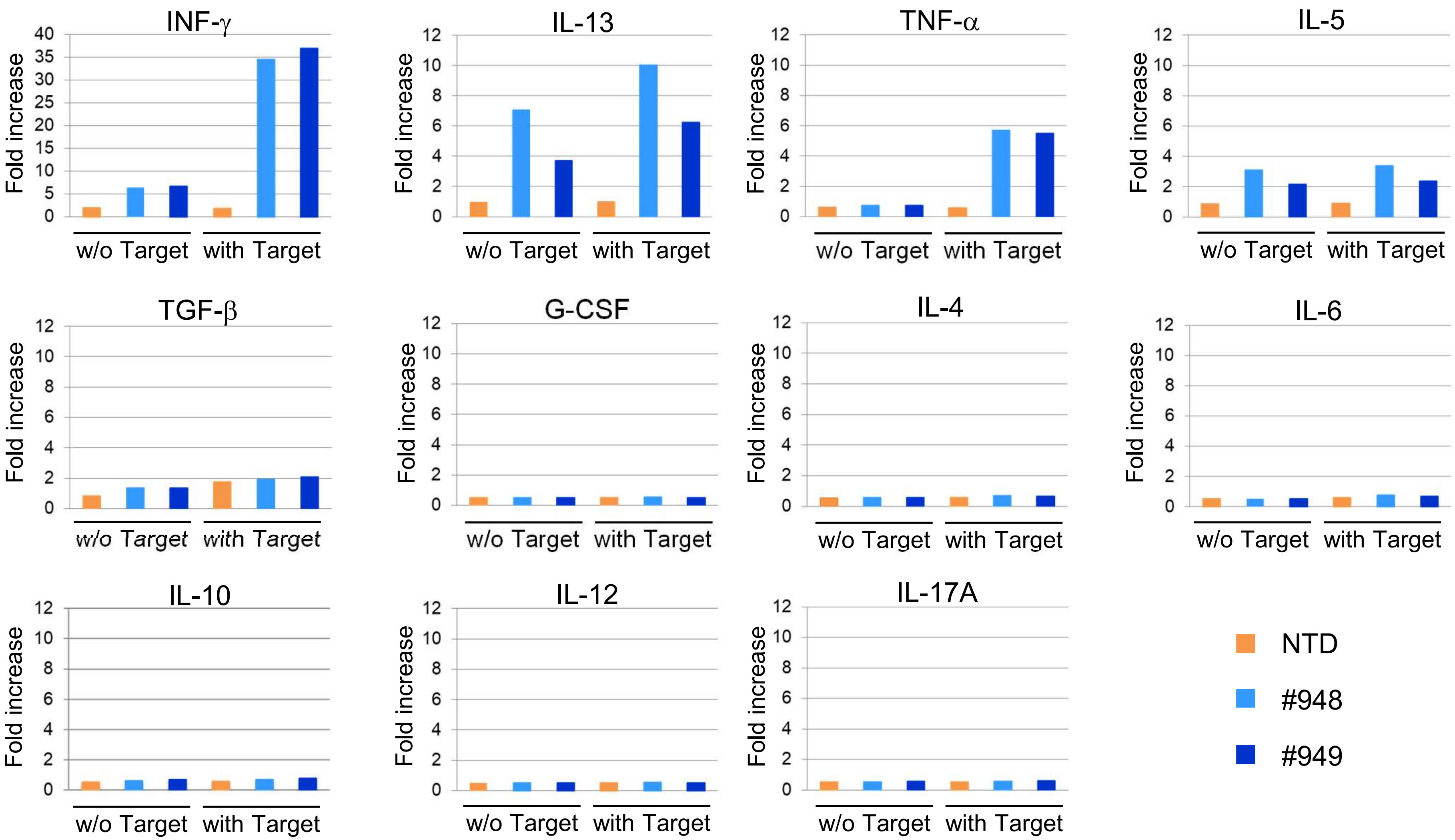

Supplementary Figure 6. Analysis of cytokine expression in the culture supernatants of T-cells expressing the MET-CAR co-cultured or not with Target cells (EBC-1). Graphs represent fold increase in the amount of each cytokine versus not transduced T cells cultured in the absence of Target cells. For each cytokine, the value of the negative CTRL is reported as 1. Values below 2 are considered below the level of detection. NTD: Not transduced T-cells; #948 and #949: MET-CAR expressing T cells. INF: Interferon; TNF: Tumor Necrosis Factor; IL: Interleukine; TGF: Transforming Growth Factor; G-CSF: Granulocyte Colony Stimulating Factor.

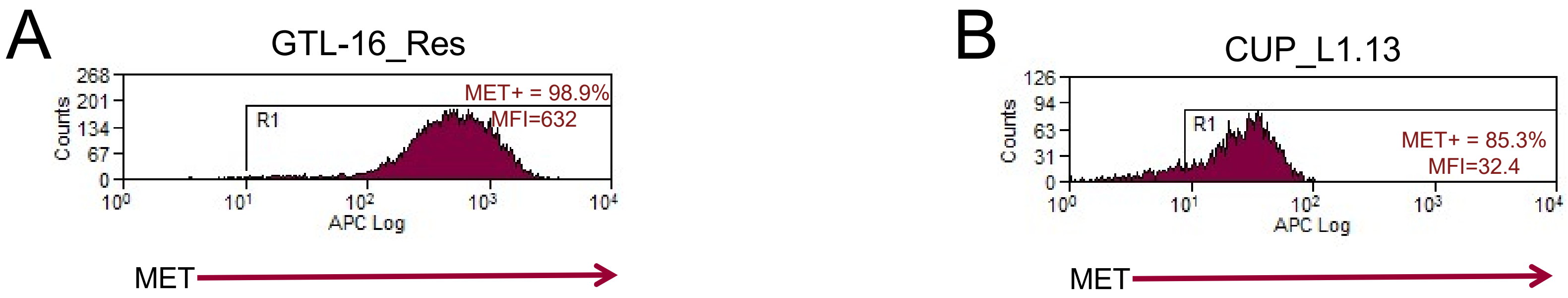

Supplementary Figure 7. MET expression on carcinoma cells featuring MET gene amplification and overexpression resistant to anti-MET agents. (A) Analysis of surface MET levels by flow-cytometry on GTL-16_Res gastric carcinoma cells. (B) Analysis of surface MET levels by flow-cytometry on L1.13 primary CUP cells. Percentage of MET positive cells and Mean Fluorescence Intensity (MFI) are indicated in the plots. Counts: number of the detected events.

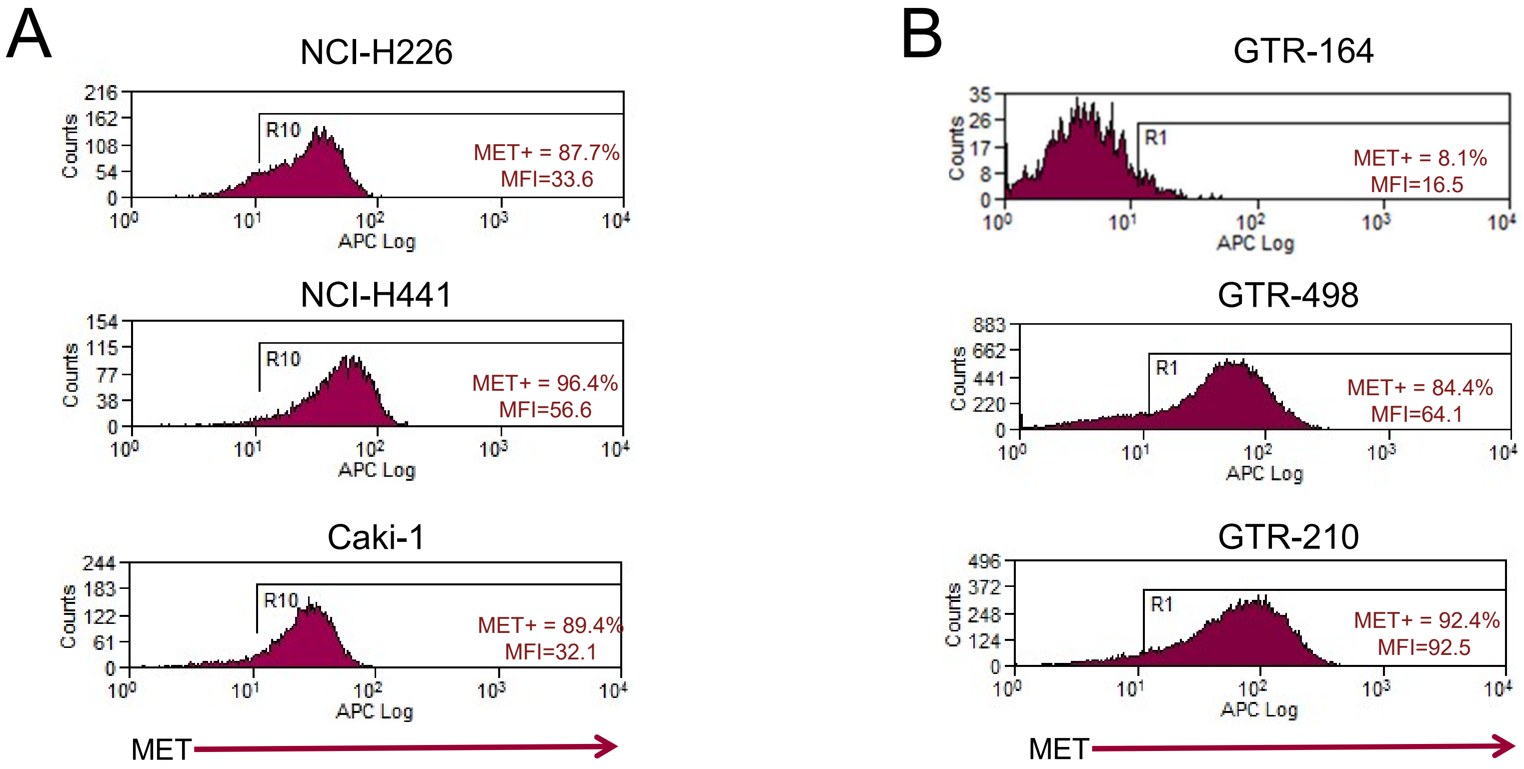

Supplementary Figure 8. MET expression analyzed on carcinoma cells featuring MET overexpression due to transcriptional upregulation of the MET gene, present as diploid or at low copy number. (A) Analysis of surface MET levels by flow cytometry on carcinoma cell lines. (B)) Analysis of surface MET levels by flow cytometry on Gastric carcinoma primary cells derived from human tumors grown in immunodeficient mice (PDX). Percentage of MET positive cells and Mean Fluorescence Intensity (MFI) are indicated in the plots. Counts: number of the detected events.
